# Blood vessel and osteocyte networks concurrently rearrange during bone maturation and decline during aging in the femur of male mice

**DOI:** 10.18632/aging.206302

**Published:** 2025-08-22

**Authors:** Mathilde Palmier, Marlène Maître, Hélène Doat, Thierry Lesté-Lasserre, Claudine Boiziau, Delphine B. Maurel

**Affiliations:** 1 Inserm, University of Bordeaux, BioTis Laboratory UMR 1026, Bordeaux 33000, France; 2 Inserm, University of Bordeaux, Neurocentre Magendie UMR 1215, Bordeaux 33000, France

**Keywords:** bone development, physiological aging, osteocyte differentiation, osteopenia

## Abstract

While blood vessels and osteocytes have been studied independently, their simultaneous changes with age remain undescribed. Our objective was to investigate the age-related evolution of both osteocyte and blood vessel networks in mouse cortical bone, and to assess the associated effects on osteocyte markers and oxygen intracellular levels. We analyzed femurs of male Flk1-GFP mice from growing, mature, middle-aged, and aged groups with techniques such as laser microdissection followed by RT-qPCR, tissue clearing and 3D fluorescence imaging. In the mature animals - when the cortical bone was thicker than in the growing animals - the osteocyte density, the number of dendrites per osteocyte and the blood vessel density were lower. This was associated with a reduced expression of *Pdpn* and with a smaller fraction of osteocytes exhibiting low intracellular oxygen. In aged animals - when cortical bone was thinner than in mature animals - the number of dendrites per osteocyte and the blood vessel density were lower. This was associated with a reduced *Gja1* (Cx43) expression. Our results suggest that changes in the osteocyte network during maturation and aging are led by distinct mechanisms, and that the cortical bone blood vessels are not the main source of oxygen for osteocytes.

## INTRODUCTION

From birth, bone mass continuously increases until it reaches its peak, and then it starts to decrease with aging. During growth, the morphology of long bones changes, they lengthen at the extremities (epiphyses) while the center part (diaphysis) becomes wider to maintain stability and strength. Growth in width occurs through the process of continuous bone matrix apposition at the periosteal side [1, 2]. It is also characterized by the modeling drift making the shape asymmetrical [3]. Immature bone is progressively replaced by mature bone, and later remodeling occurs to replace old bone. This process maintains bone mass and quality, and enables its adaptation to mechanical loads [4].

In aged individuals, bone remodeling is preponderant and dysregulated, with resorption being higher than formation leading to a thinning of the cortical bone [5, 6]. In the most severe cases, it evolves towards osteoporosis with characteristics such as decreased bone density and increased intracortical porosity that leads to fractures [7, 8]. To understand how pathology evolves, what are the precursor signs and when the bone starts to weaken, it is essential to first understand the actors and events occurring in physiological conditions throughout life.

Osteocytes represent more than 95% of the bone cells and are terminally differentiated osteoblasts that became embedded in their mineralized matrix. While differentiating, they expand their dendrites to reach other cells and to form a network. They express distinct markers depending on their stages of maturity [9]. In growing bone, Youlten et al. showed that they have a specific transcriptome in comparison with mature, adult bone [10]. With aging, Tiede Lewis et al. showed that the osteocyte network declines, with a lower cell density and a loss of dendrites per cell [11]. Yilmaz et al. also characterized this decline in a prematurely aged PolgA mouse model [12]. As osteocytes orchestrate bone remodeling [9], these changes need to be better investigated to understand their role. To our knowledge, osteocyte gene expression and their morphology have been mostly studied independently and there is no clear view of the differences between maturation and aging.

Blood vessels share the same environment as osteocytes. In rodents, blood vessels directly connect the bone marrow cavity to the periosteal surface [13]. Prisby et al. showed that the blood flow was decreased with aging in the marrow cavity and in the metaphysis, but not in the cortical bone [14]. Moreover, Grüneboom et al. showed that the trans-cortical blood vessel number decreased with aging [15]. However, more studies focusing on the blood vessels inside the cortical bone need to be done to understand their roles.

Osteocytes and blood vessels are essential for bone homeostasis; they are in direct contact and evolve in the same changing environment. Separate investigations have been devoted to these networks, but their concomitant evolutions with age have not been described. We propose to analyze their chronology of changes in bone gain and bone loss contexts, namely maturation and aging, to identify potential interdependences. Previous studies suggested that osteocyte metabolism and viability are impacted by changes in the blood vessel network in cortical bone [16, 17]. Therefore, we hypothesized that osteocyte access to oxygen would depend on the blood vessel network density.

We used C57Bl/6 Flk1-GFP male mice. This model expresses the GFP in blood vessels and this strain was shown before as suitable for aging-related osteopenia studies [18, 19]. We adapted tools, previously described by Tiede-Lewis et al. [11] and Grüneboom et al. [15], to specifically and directly study osteocytes and blood vessels. It enabled us to label, image and analyze in 3D osteocytes and blood vessels. In addition, we collected osteocytes thanks to a laser microdissection protocol previously established in our group [20] in order to specifically analyze their gene expression. We confirmed the protein synthesis in osteocytes through immunofluorescence.

## RESULTS

We studied the femur diaphysis of mice aged from 5 weeks to 24 months. We compared bones from 5-week-old, still growing animals with adult mature mice (5–8-month-old), representing the analysis of the maturation phase. In parallel, the aging process was studied by comparing the same mature bones with bones from middle-aged (15–18-month-old) and aged (21–24-month-old) animals.

### Cortical bone thickness during bone maturation and aging

**Figure 1:**
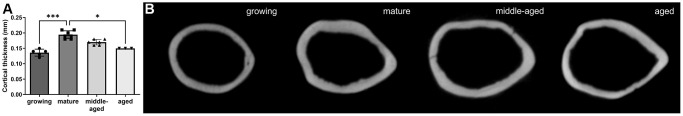
**MicroCT analysis of the femur cortical thickness at midshaft, for the 4 age groups.** (**A**) Comparison of the cortical bone thickness at mid-diaphysis between the 4 groups. (**B**) Transversal microCT images of the femur mid-diaphysis of mice from the 4 age groups. The data are presented as mean ± s.d. Statistical analyses: KW, Dunn’s multiple comparison test with the mature group as reference, ^*^p < 0.05, ^***^p < 0.001.

To validate the maturation and aging model in the Flk1-GFP mice, we verified that we obtained the expected morphological changes in bone [19]. MicroCT analysis on a length of 500 μm enabled us to study the changes in femoral bone diaphysis geometry as a function of age. In Figure 1A, we show the evolution of the cortical bone thickness with age, illustrated with 2D images in Figure 1B. The mature animals had a larger cortical thickness at mid-diaphysis than the growing (*p* < 0.001) and the aged animals (*p* < 0.05). The cortical thickness increased by 0.06 mm (+ 43%) over the 4–7 months of maturation and decreased by 0.05 mm (−26%) over the 13–16 months of aging. The evolution of the medullary cavity volume, the periosteal perimeter and the bone volume can be found in Supplementary Figure 1: the volume of medullary cavity and the periosteal perimeter increased with age (r_s_ = 0.85, and r_s_ = 0.75, *p* < 0.0001); in contrast, no correlation between cortical bone volume and age was observed due to a high increase during maturation (+ 75%, *p* < 0.001) followed by non-significant changes during aging.

### Blood vessel density in cortical bone

**Figure 2:**
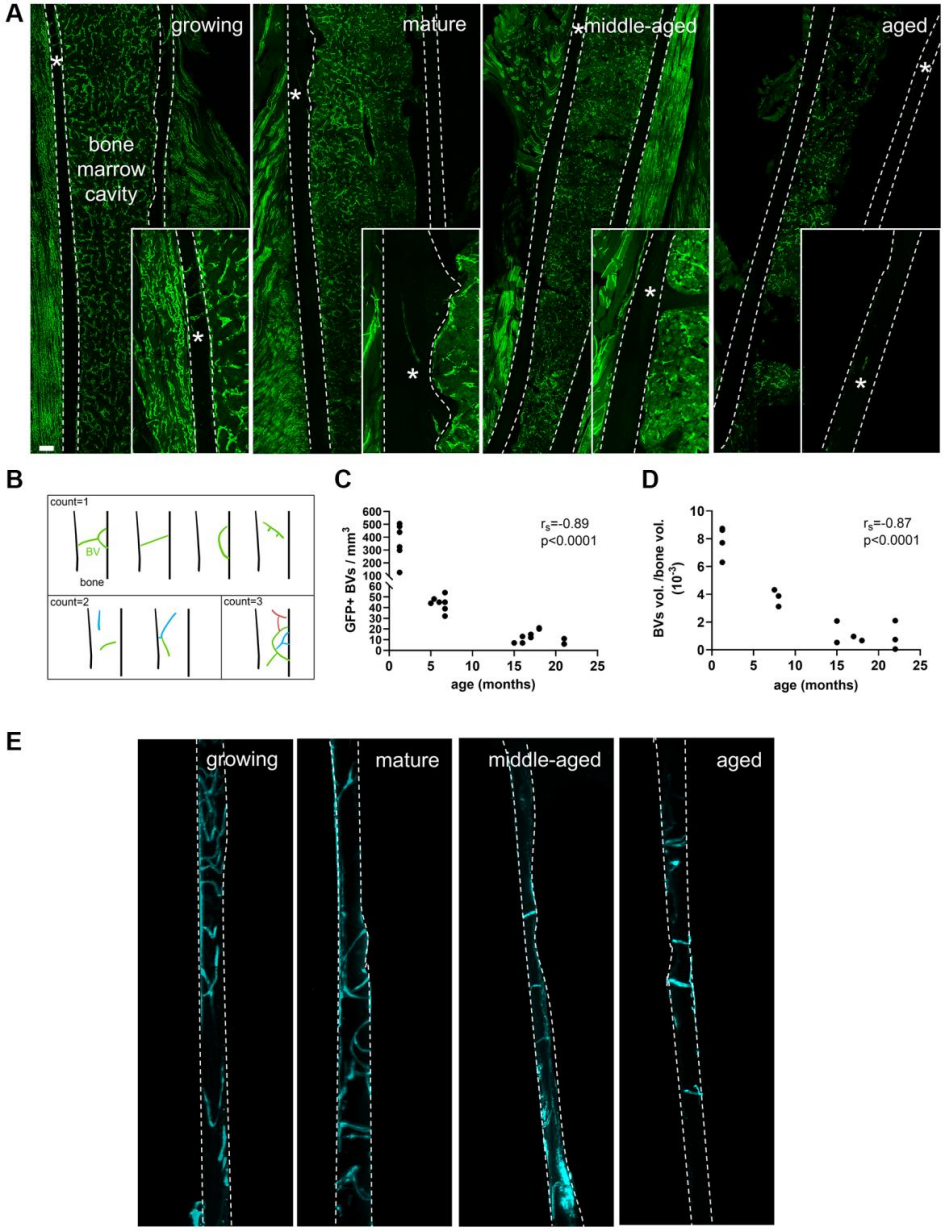
**Blood vessel (BVs) network characterization.** (**A**) GFP^+^ (green) blood vessels in growing, mature, middle-aged and aged femurs, scale bar = 200 μm. (**B**) Categorization of the branched vessels used for quantification. (**C**) Correlation between the number of GFP^+^ BVs per bone volume and age (months). (**D**) Correlation between the percentage of volume of BVs per mm^3^ of cortical bone and age (months). (**E**) Lectin^+^ (cyan) blood vessels in cortical bone in growing, mature, middle-aged and aged femurs. ^*^ indicates the blood vessel location. The dashed lines delineate bone. Statistical analyses: Spearman’s correlation.

We quantified the number of GFP positive blood vessels (Figure 2A) per mm^3^ of cortical bone (70-μm-thick sections) in the Flk1-GFP mice [18]. In Supplementary Figure 2, we verified the co-localization of GFP and CD31 in each group of age. Since we observed branched blood vessels in the growing animals, we used the categorization shown in Figure 2B to perform the quantification. The analysis showed that the number of blood vessels per mm^3^ was negatively correlated with age (r_s_ = −0.89, *p* < 0.0001, Figure 2C). To perform an analysis more representative of the network, additional animals from the same age groups were injected with lectin (a marker of capillary endothelium) conjugated to a fluorophore. The cortical bone was made transparent through tissue clearing. We could observe and segment for analysis the capillaries embedded in the bone matrix as shown in Figure 2E and in the animation in Supplementary Video 1. We quantified the volume of lectin-bound vessels per volume of bone and found that it was negatively correlated with the age of the animals (r_s_ = −0.87, *p* < 0.0001, Figure 2D). It was divided by 2.1 between the growing and the mature stages and then, by 3.6 between mature and middle-aged stages.

### Osteocyte network density

**Figure 3:**
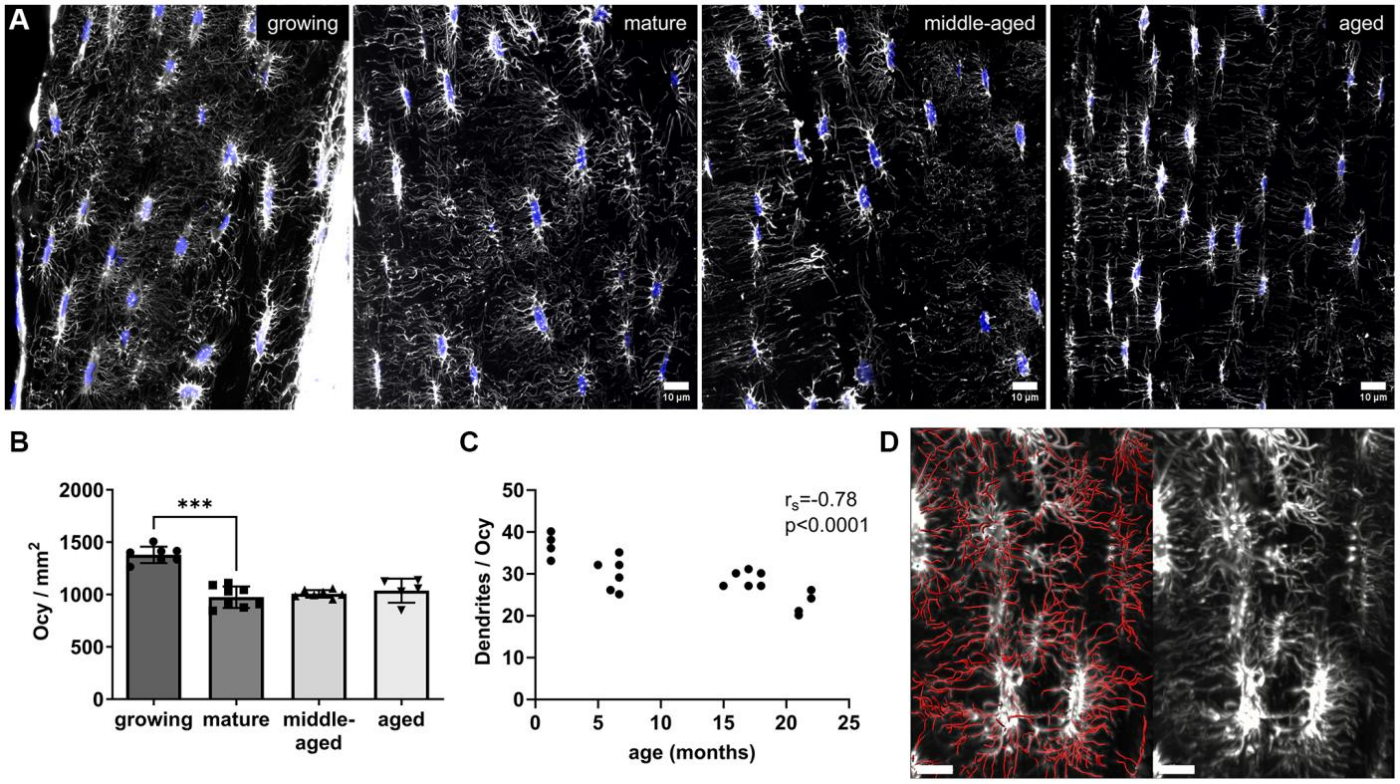
**Osteocyte network age-related changes.** (**A**) Representative images of the osteocyte network labeled with phalloidin (white) and nuclei (in blue). (**B**) Number of osteocyte nuclei (Ocy) per mm^2^ of bone (10-μm-thick section) for each group. (**C**) Correlation between the number of dendrites per osteocyte and age (months). (**D**) Modelization (in red) of the dendrites used for quantification of the number of dendrites per osteocyte (scale bar = 10 μm). The data are presented as mean ± s.d. Statistical analyses: KW, Dunn’s multiple comparison test with the mature group as reference, Spearman’s correlation, ^***^p < 0.001.

After analyzing the blood vessel network, we characterized the concurrent changes in morphology of the osteocyte network. We used DAPI to quantify nuclei and phalloidin to quantify the dendrites (Figure 3A). The osteocyte network underwent strong changes during maturation. On equivalent surface areas of bone sections, the mature bone had about 29% less osteocytes per mm^2^ than the growing bone (*p* < 0.001 Figure 3B). After reaching maturity the osteocyte density in bone was not further affected by aging. Indeed, no significant changes were observed with the middle-aged and aged animals compared to the mature ones (Figure 3B). However, a regular decrease in the number of dendrites per osteocyte was observed with age, showing a strong negative correlation with the age of the animals (r_s_ = −0.78, *p* < 0.0001, Figure 3C). An example of the modelization (in red) of the dendrites used for quantification of the number of dendrites per osteocyte is shown in Figure 3D. In addition to this global evolution, a high heterogeneity in the organization of the osteocyte network was noted at all studied ages, consistent with the presence of woven bone at any age described by Ferguson et al. [19]. In well-organized areas, we observed aligned osteocytes and dendrites heading towards the bone surface while in other areas, there were disorganized osteocytes without common alignment of their bodies or dendrites as shown in the Supplementary Figure 3.

### Expression of markers associated with the osteocyte network

**Figure 4:**
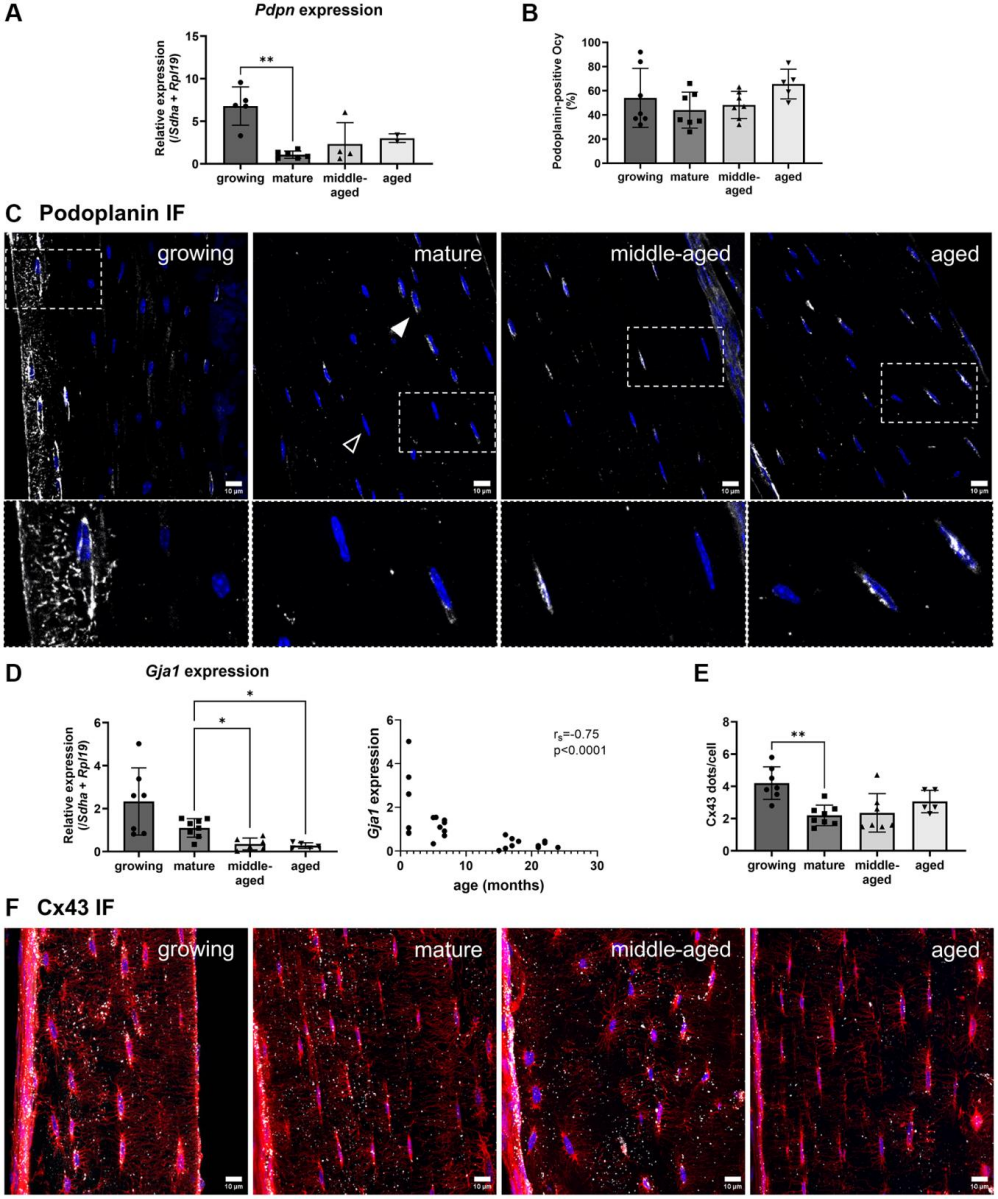
**Age-related changes for the gene expression and protein synthesis of Podoplanin and Connexin 43.** (**A**) Podoplanin gene expression. (**B**) Quantification of Podoplanin-positive osteocytes (Ocy) based on immunofluorescence (IF). (**C**) Representative images for Podoplanin immunofluorescence for the 4 groups. Podoplanin labeling appears in white (filled arrowhead), the empty arrowhead shows a negative cell, and nuclei appear in blue. (**D**) *Gja1* gene expression and correlation with age (months). (**E**) Quantification of dots/cell body based on immunofluorescence. (**F**) Representative images for Connexin 43 immunofluorescence, Connexin 43 labeling appears in white, nuclei appear in blue. The data are presented as mean ± s.d. Statistical analyses: KW, Dunn’s multiple comparison test with the mature group as reference, Spearman’s correlation, ^*^p < 0.05, ^**^p< 0.01.

As age affects the number of dendrites per osteocyte (Figure 3C), we investigated the changes in network markers in osteocytes isolated thanks to laser microdissection. Laser-assisted microdissection enabled us to specifically collect osteocytes in cortical bone to subsequently analyze their mRNA with minimal contamination by other cell types [20]. We analyzed the *Pdpn* (coding for Podoplanin/E11) and *Gja1* (coding for Connexin 43, Cx43) expressions. Podoplanin is involved in dendrite elongation [21] and Connexin 43 is either present on dendrites as a gap junction for cell-cell communication, or on the cell bodies as hemi-channels [22]. The analysis of *Pdpn* showed that its expression was 6 times higher at the growing stage than at the mature one (Figure 4A, *p* < 0.01). However, its expression remained unchanged with aging (in the middle-aged and aged groups, the gene expression was detected only for part of the samples, Figure 4A). Immunofluorescence analysis revealed Podoplanin-positive cells in all groups without difference (Figure 4B, 4C). Then, we found that the *Gja1* expression in the osteocytes from the middle-aged and aged animals was a third of the expression in the mature animals (*p* < 0.05, Figure 4D) and negatively correlated with age (r_s_ = −0.75, *p* < 0.0001, Figure 4D). Regarding the immunofluorescence analysis, the quantitative analysis of the number of dots per cell body showed that it was significantly higher in the growing group than in the mature, but no difference was observed with aging (*p* < 0.01, Figure 4E). Representative images of Connexin 43 immunoreactive clusters quantified as dots (in white) on the osteocyte body (in red) are shown in Figure 4F and in the animation (Supplementary Video 2). A lower number of dendrites was associated with a lower *Pdpn* expression, in the case of maturation, and with a lower *Gja1* expression during aging.

### Expression of markers associated with the osteocyte maturity and function

In addition to its role in the extension of the osteocyte dendritic network, Podoplanin is also expressed during the early stages of osteoblast-to-osteocyte transition and serves as a marker of immature, embedding osteocytes [21]. The presence of highly Podoplanin-positive cells near the bone surface in growing animals (Figure 4C) suggests active osteocyte differentiation.

**Figure 5:**
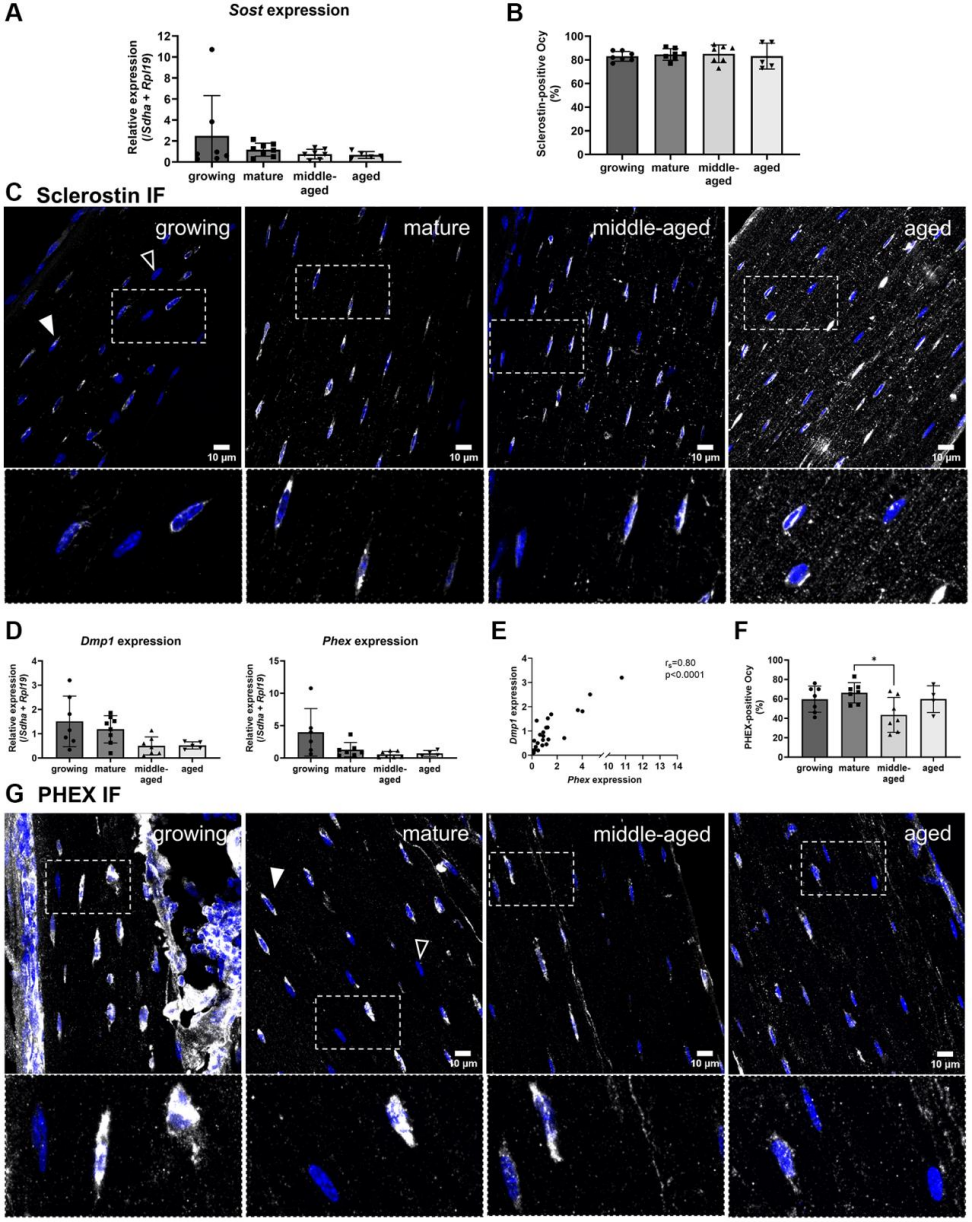
**Age-related changes for the gene expression and protein synthesis of Sclerostin, DMP1 and PHEX.** (**A**) *Sost* gene expression. (**B**) Quantification of Sclerostin-positive osteocytes (Ocy) based on immunofluorescence. (**C**) Representative images for Sclerostin immunofluorescence for the 4 groups, Sclerostin labeling appears in white (filled arrowhead), the empty arrowhead shows a negative cell, nuclei appear in blue. (**D**) *Dmp1* and *Phex* gene expressions. (**E**) Correlation between *Dmp1* and *Phex* expression in osteocytes from growing, mature, middle-aged, aged animals. (**F**) Quantification of PHEX-positive osteocytes based on immunofluorescence during aging. (**G**) Representative images for PHEX immunofluorescence for the 4 age groups, PHEX labeling appears in white (filled arrowhead), the empty arrow shows a negative cell, nuclei appear in blue. The data are presented as mean ± s.d. Statistical analyses: KW, Dunn’s multiple comparison test with the mature group as reference, ^*^p < 0.05.

To examine how osteocyte differentiation progresses and is maintained from bone maturation through aging, we analyzed their gene expression after isolation using laser-assisted microdissection. While differentiating, the cells start to express mineralization markers such as Dmp1 and Phex and eventually, mature osteocytes express and secrete Sclerostin, which inhibits bone formation [23, 24]. The expression of *Sost* (coding for Sclerostin) remained unchanged with age (Figure 5A). We also quantified the proportion of Sclerostin-positive cells after immunofluorescence (Figure 5B). Most embedded cells expressed Sclerostin (in white) across all ages, with an apparent increase in signal intensity within the lacuno-canalicular network of aged animals (Figure 5C).

The expressions of *Dmp1* and *Phex* did not vary significantly with maturation and aging (Figure 5D), but their expressions were positively correlated (Figure 5E). Additionally, the percentage of PHEX-positive osteocytes (in white) in the middle-aged animals was lower than in mature animals (*p* < 0.05, Figure 5F, 5G).

### Oxygen levels in osteocytes

**Figure 6:**
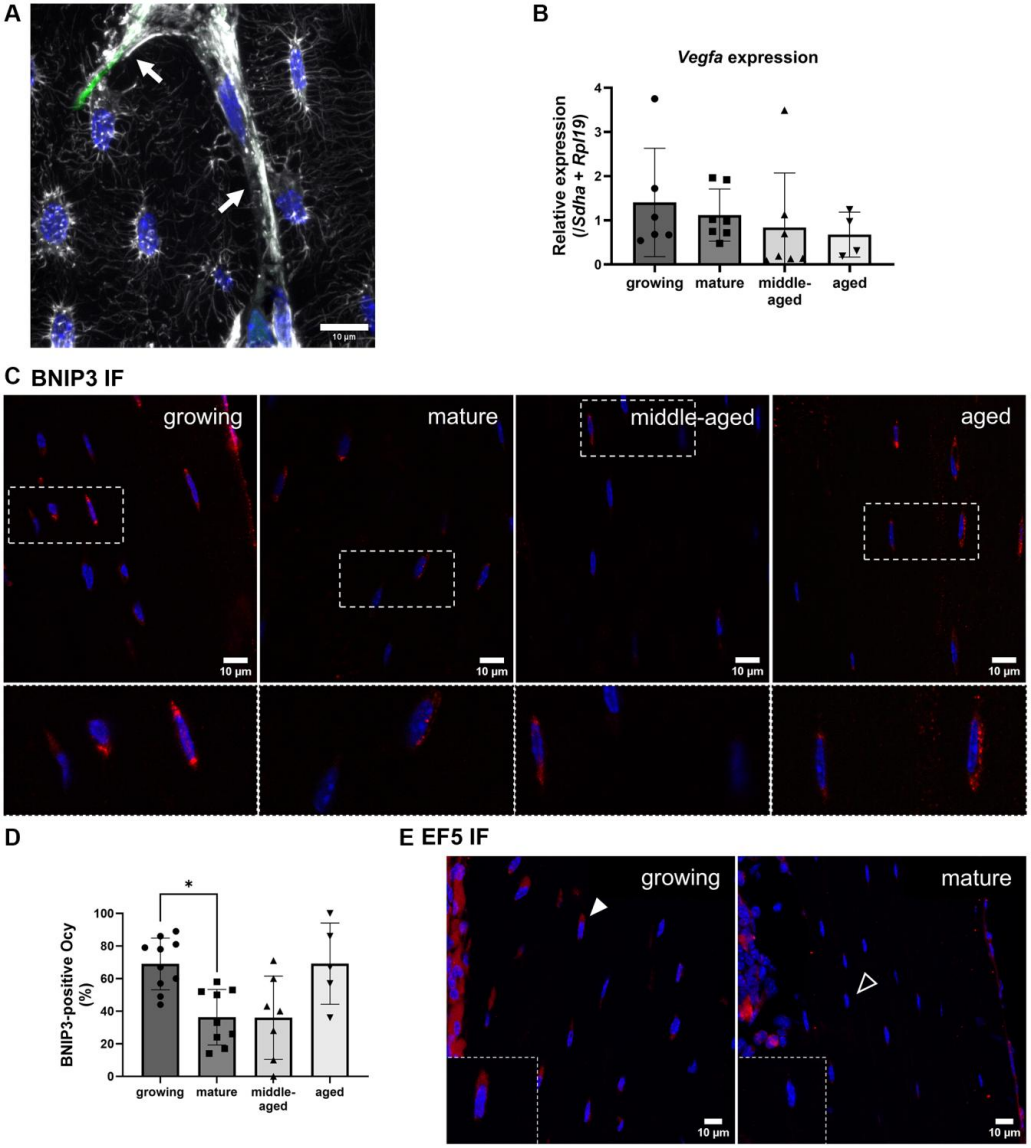
**Hypoxia detection and VEGFA expression in osteocytes.** (**A**) Phalloidin labeling in a growing animal showing numerous connections between osteocyte dendrites (white) and blood vessels (arrows). (**B**) *Vegfa* gene expression. (**C**) Representative images for BNIP3 immunofluorescence for the 4 groups, BNIP3 labeling appears in red, nuclei appear in blue. (**D**) Quantification of BNIP3-positive osteocytes (Ocy) based on immunofluorescence (IF). (**E**) Representative images for EF5 detection for the growing and mature groups, positive cells appear in red (filled arrowhead), the empty arrowhead shows a negative cell, nuclei appear in blue. The data are presented as mean ± s.d. Statistical analyses: KW, Dunn’s multiple comparison test with the mature group as reference, ^*^p < 0.05.

The staining of actin cytoskeleton revealed numerous connections that exist between osteocyte dendrites and blood vessels as shown in Figure 6A and Supplementary Video 3. Blood vessels are generally thought as cell supplier in oxygen and nutrients and previous work has pointed to a link between blood vessel embolism and osteocyte viability [16]. Therefore, we hypothesized that blood vessels supply osteocytes in oxygen and the oxygen level of osteocytes would depend on the blood vessel density.

Since VEGFA is a growth factor generally used to stimulate angiogenesis and its expression is driven by hypoxia [25], we assessed if the modulation of its expression could be evidenced. We found that the *Vegfa* gene expression in osteocytes was unchanged with age (Figure 6B).

To estimate the oxygenation state of osteocytes, we performed an immunolabeling of BNIP3 (Figure 6C). This protein is indeed positively regulated by HIF-1α and detected on mitochondria during hypoxia [26]. We found a higher percentage of BNIP3-positive osteocytes in the growing than in the mature group (*p* < 0.05, Figure 6D). We concluded that more osteocytes had lower access to oxygen in the growing than in the mature group.

Based on Figure 2C, the blood density was the highest in the growing group, therefore we did not expect lower oxygen levels than in the mature group. To confirm this unexpected finding, we used the EF5 compound that binds to cells depending on their intracytoplasmic oxygen level. The lowest the level of oxygen, the highest is the binding to the cells. After fluorescent labeling, cells with the lowest oxygen level are the most fluorescent. We compared fluorescence in growing and mature groups. Even though the fluorescence signal was generally low, we mainly found osteocytes labeled along the diaphysis of the growing animals (Figure 6E). Thus, our results suggest that oxygen levels in osteocyte depends on the age rather than on the blood vessel network density.

## DISCUSSION

We examined the morphology of the osteocyte network alongside specific osteocyte gene expression, while simultaneously analyzing the cortical bone blood vessel network, to compare changes occurring during maturation and aging, in male mice. We performed this study using novel and diverse techniques to specifically study matrix-embedded osteocytes and blood vessels, such as laser microdissection, tissue clearing and 3D fluorescence imaging. We showed that a decrease in osteocyte density and of the number of dendrites per osteocyte were not necessarily deleterious and were associated with increased cortical thickness during bone maturation. Additionally, we found that maturation was associated with a reduced expression of *Pdpn* and aging, with a reduced expression of *Gja1*, both in accordance with fewer dendrites per osteocyte.

Overall, although maturation and aging (in a healthy situation) have opposite outcomes on cortical bone thickness, they were both accompanied by dendrite and blood vessel loss in cortical bone. Additionally, we observed that the changes during aging were milder than those occurring during maturation.

Finally, osteocytes are regularly mentioned as hypoxic cells. Here we show that not all of them have low oxygen levels, and our results suggest that it depends on the age and not on the trans-cortical blood vessel density.

We started our study with the validation of the maturation and aging model in the Flk1-GFP mice, verifying the expected morphological changes in bone [4, 19]. We observed an increase in cortical thickness during maturation, and the thinning of the cortical bone during aging. This is consistent with growth and osteopenia.

During maturation, the blood vessel and osteocyte networks concurrently rearrange. It was characterized, on the one hand, by a substantial decrease in blood vessel density, which can be assimilated to vascular remodeling during growth [27], and on the other hand, by a decrease in osteocyte density and number of dendrites per cell. It is well established that osteocytes control bone adaptation to mechanical cues by detecting these signals through molecular mechanosensors on their cell bodies and dendritic processes. The changes in osteocyte network with maturation may be attributed to the distinct mechanosensory requirements between growing and mature bone tissue [28, 29]. During long bone growth, new bone matrix must be formed while immature bone undergoes remodeling. This simultaneous need to strengthen multiple regions [4, 30] likely increases the demand for mechanosensitivity, hence a high need for connections between osteocytes through a dense network. Moreover, Cx43 hemi-channels on osteocyte bodies have been shown to open with mechanical load for the rapid release of prostaglandin E2 [31]. In our study, we found a higher number of Cx43 hemi-channel clusters in growing animals. This is in accordance with the necessity of a higher capacity to sense mechanical load.

During aging, the blood vessel and osteocyte networks concurrently declined, which is consistent with previous studies. Tiede Lewis et al. studied the osteocytes network in the femur of C57Bl/6 male and female mice using imaging techniques [11]. Our findings are consistent, with the exception that we did not detect any significant change in osteocyte density. Regarding blood vessels, Grüneboom et al. showed that the trans-cortical vessel number decreased with aging in the tibiae of DBA/1 DEREG mice [15].

Then, we examined how osteocyte differentiation progresses and was maintained from bone maturation through aging. Embedding osteocytes express *Pdpn*, differentiating further, they express *Dmp1* and *Phex* and eventually, mature osteocytes express *Sost* [24].

We detected Podoplanin in recently embedded cells located near the bone surface in growing animals, accompanied by higher *Pdpn* expression levels compared to mature animals. Although the proportion of Podoplanin-positive cells was similar across all groups, cells expressing Podoplanin along the bone surface were observed exclusively in growing animals (Figure 4B, 4C). This could indicate ongoing differentiation in growing animals and network maintenance in aging ones.

*Dmp1* and *Phex* expressions were positively correlated confirming that they have related expression patterns. This was demonstrated in another study showing the similar effect of *Dmp1* and *Phex* deletions on FGF23 regulation [32]. Moreover, PHEX was detected in all groups, meaning that osteocytes retain this marker regardless of age, supporting its role in maintaining osteocyte function.

Regarding the Sclerostin analysis, we found a high proportion of positive osteocytes in all the groups, without any difference. We also did not find any change in *Sost* gene expression with age. Thompson et al. also observed Sclerostin-positive and Sclerostin-negative subpopulations but they reported an increase of Sclerostin-positive osteocytes in cortical bone in aged mice femur [33]. The difference between the two studies could be explained by the fact that they studied a C3H/HeJ mouse strain and it was shown that there is a different response to aging between the mice strains [34]. In their study, the percentages of Sclerostin-positive osteocytes were lower than the percentages that we found. In our images, we noticed different levels of intensity among the osteocytes in each section and among the groups. The osteocytes that were quantified by Thompson et al. could correspond to one subset of highly expressing osteocytes in our experiments.

Finally, we observed differences in oxygen levels in osteocytes, unrelated to blood vessel density in cortical bone. We found a higher proportion of BNIP3 positive osteocytes in the growing group than in the mature group. Interestingly, the percentage of BNIP3 positive osteocytes corresponds to the percentage of osteocytes stained with pimonidazole (~70%) found by Wu et al. in the tibia of 8-week-old mice [35]. In this study, we qualitatively confirmed the presence of cells with lower oxygen levels in growing animals with EF5 injection. This could have also been confirmed by quantification of HIF-1α immunolabeling in growing animals. It has been shown that the stabilization of HIF-1α isoforms in osteoblasts and osteocytes contributes to the increase of cortical bone thickness during skeletal development [36, 37]. The lower oxygen levels observed in osteocytes during bone development in growing animals, compared to mature animals, may reflect a physiological requirement associated with bone growth. This is supported by the fact that Prideaux et al. showed that osteocytes rely on glycolysis during *in vitro* differentiation [38].

A high percentage (69 ± 25%) of osteocytes in the aged group also presented BNIP3 positive puncta. Since BNIP3 is involved in autophagy of mitochondria (“mitophagy”), we suggest that BNIP3 immunolabeling might be a marker of active mitophagy. In certain organs such as healthy muscles, although expressed continuously, BNIP3 shows an increased expression during aging as a process to mitigate inflammation and prevent sarcopenia [39]. This hypothesis should be tested using tools such as the mito-Keima protein (targeted to mitochondria and whose fluorescence properties change when the protein is in the acidic medium of lysosomes) [40].

As *Vegfa* expression was unchanged, we did not find any evidence of communication between osteocytes and blood vessels. In a recent study, Liao, Chen, Zhou et al. could not find any indirect regulation of endothelial cells by osteocytes in growing animals but they demonstrated a direct transfer of mitochondria from osteocytes to endothelial cells through the dendrites [41].

While the current findings do not conclusively establish the role of oxygen supply regulation in osteocytes, they indicate that intracellular oxygen levels are not influenced by the density of the surrounding vascular network.

Despite its assets, this study presents some limitations. First, in the present work we favored the number of groups to have a larger view on bone physiological changes, in particular by studying two groups of aging animals; this led us to study only male mice, although some differences might be observed with females taking into account the sexual dimorphism [42]. Then, even though the laser microdissection technique is a relevant tool to specifically isolate osteocytes, the mRNA amount collected allows the study of only a limited number or well-expressed osteocyte genes, thus genes like *Fgf23* could not be detected. Eventually, this work explored a limited aspect of the interdependences between blood vessels and osteocytes, which need to be further explored.

In conclusion, we found that the osteocyte network was denser in the growing group than at any other older age, presumably to adapt to the mechanical load. By studying the maturation process, we found that a decrease in blood vessel and osteocyte densities, and in dendrite number are not always associated with bone loss. It means that studying aging with control animals younger than the age of bone maturity is not relevant. Additionally, we found that the osteocyte intracellular oxygen level was not influenced by the density of the surrounding vascular network in cortical bone. By studying aging, we observed a decrease in blood vessel density with maintained osteocyte density. This could suggest that the blood vessels in the cortical bone are not the main source of oxygen for osteocytes. In fact, osteocytes could be mainly supplied by blood vessels from the periosteum and the endosteum through the interstitial fluid. This work gives a clearer view on how osteocytes and blood vessels concurrently rearrange during bone maturation and decline during physiological aging.

## MATERIALS AND METHODS

### Animals

Male heterozygous Flk1-GFP mice were used [18]. The lineage has been maintained by breeding with WT C57Bl/6J females for at least 10 generations in the animal facility at the University of Bordeaux (accreditation number A33-063-917), and the project received the authorization of the French Ministry of Higher Education, Research and Education (APAFIS #32816-2021083017472443 v4, APAFIS#27986-2020110911537075 v5). Four age groups were compared: a stage of skeleton development “growing” (5 weeks old), and three stages of adulthood “mature” (5–8 months old), “middle-aged” (15–18 months old) and “aged” (21–24 months old). The group sizes were estimated according to the literature [11] and to give a power of 80% in the statistical analysis. Fifty animals were used in this study. The animals were housed together in cages in an enriched environment (houses and nesting) with a 12-hour day/night cycle and food (A03 breeding/A04 maintenance diets, SAFE, Augy, France), and water *ad libitum*.

### Bone sample preparations

#### Preparation A: bone samples for hypoxia detection, 3D blood vessel network visualization and microCT

The animals (growing *n* = 7, mature *n* = 7, middle-aged *n* = 5, aged *n* = 3) were injected with 10 μL/g of a 10 mM solution of EF5 (EF5 Hypoxia Detection Kit CS222743, Merck, Fontenay sous Bois, France) intraperitoneally, 2 h before euthanasia. Fifteen minutes before euthanasia, we performed either a 100 μL intravenous injection in the tail of adult animals, or a 50 μL retro-orbital injection in the sinus of growing animals with lectin coupled to a fluorophore (Lycopersicon Esculentum Tomato Lectin, DyLight 649, 1 mg/mL, Invitrogen by Thermo Fisher Scientific, Illkirch, France). Ten minutes after the injection, an intraperitoneal dose of xylazine (Rompun, 20 mg/kg) and ketamine (Imalgene 1000, 100 mg/kg) was administered before intracardiac perfusion of 20–30 mL of PBS 1X followed by 20–30 mL of 4% paraformaldehyde (AntigenFix, # P0016, Diapath, Martinengo, Italy). The femurs were excised, and the bone marrow was flushed (syringe 26G and PBS 1X). The femurs were immersed in paraformaldehyde 4% for 24 h at 4°C. One femur per animal was decalcified and cryopreserved as follows: it was first transferred in EDTA 10% in PBS 1X, pH 7.4 for 1 week, then it was placed in sucrose 15% in PBS 1X for one day, and in sucrose 30% in PBS 1X overnight. The next day, it was embedded in OCT, Optimum Cutting Temperature gel (TFM-C, MM France, Brignais, France) and stored at −80°C until cryosections were done for subsequent analyses. The other femur underwent a clearing protocol as described by Grüneboom et al. 2019 [15]. It was dehydrated in 50% then 70% and 100% ethanol twice for 24 h at 4°C. Finally, it was placed in an ethyl cinnamate bath (98%, ACROS Organics by Thermo Fisher Scientific) overnight at room temperature before imaging (3D vascular network). Samples were stored in ethanol 100% at 4°C before microCT analysis.

#### Preparation B: bone samples for osteocyte analyses, and 2D blood vessel network visualization

Flk1-GFP mice (growing *n* = 8, mature *n* = 8, middle-aged *n* = 7, aged *n* = 5) were injected with 100 μL of Dextran Texas red 10,000 MW diluted in PBS 1X (10 mg/mL, Invitrogen) in the tail vein or in the retro-orbital sinus for growing mice, 5 min before euthanasia. The animals underwent cervical dislocation, then the left and right femurs were excised. The left femurs were fixed in 4% paraformaldehyde for 24 h at 4°C and they were transferred in 10% EDTA, pH 7.4 for 1 week for decalcification. Then, they were immersed in 15% sucrose for one day and 30% sucrose overnight for cryopreservation. Finally, they were embedded in OCT (MM France) the next day and stored at −80°C until cryosections were done. Right femurs were placed in OCT (MM France) and snap frozen in liquid nitrogen before being stored at −80°C for laser microdissection.

### Imaging of bone, blood vessel and osteocyte networks

#### MicroCT imaging

MicroCT scanning of bone samples (preparation A) was performed with a Skyscan 1276 high resolution, fast *in vivo* desktop device from Brüker (Billerica, MA, USA) in accordance with recommended guidelines [43], using an X-ray energy of 70 kV (200 μA), a voxel size of 12 μm, 3 frames average per rotation step of 0.4°, exposure time 520–550 ms, on a 180 degree range of motion, with a 0.5 mm Aluminum filter. Images were reconstructed using the NRecon software and analyzed with the CTAn software. The grayscale index (1–255) threshold was set to 78 for cortical bone midshaft, to segment mineralized tissue. The Volume of Interest (VOI) chosen for cortical bone analysis at midshaft was 42 slices (500 μm). It was contoured externally starting at the distal end of the lesser trochanter and progressing toward the knee joint. The marrow cavity was contoured internally, and the subtractive ROI mode was applied to analyze separately the cortical part and the marrow space. Bone parameters were analyzed using the CTAn software: cortical thickness (mm), cortical bone volume (mm^3^), periosteal perimeter (mm), medullary cavity volume (mm^3^).

#### 2D imaging of vascular network and analysis

Flk1-GFP mice express GFP under the control of the Flk1 (KDR/VEGFR2) promotor, making blood vessels appear with a green fluorescence. Hundred microns frozen sections (preparation B) were cut using a cryostat (CM3050S, Leica Microsystems, Nussloch, Germany), and collected on SuperFrost Plus adhesion slides (Epredia, Thermo Fisher Scientific). They were rinsed for 5 minutes with PBS 1X and then mounted with Fluoromount-G (#15586276, Invitrogen). Imaging was performed by a blinded investigator with a spinning disk confocal microscope (Leica DMi8 Yokogawa CSU-W1 + ILAS2) equipped with 491 laser line (50%), with a 20× objective. Exposure time was 200 ms (491 nm). Acquisitions were performed on a total thickness of 70 μm. Voxel dimensions were 0.55 μm (x, y) and 1 μm (z). The Fiji software was used to measure the surface of interest and then calculate the volume of analysis (0.1 to 0.2 mm^3^). The Imaris software (Bitplane V 9.0.1) was used to manually count the number of GFP positive vessels inside the cortical bone. The ratio of blood vessel/mm^3^ was then calculated.

#### 3D imaging of vascular network and analysis

Each femur (preparation A) was mounted horizontally and immersed in ethyl cinnamate solution. Imaging was done using the LaVision BioTec ultramicroscope (Bielefeld, Germany) equipped with a 647 nm laser line (50%), a sCMOS Andor camera, and a 0.5 NA 2× objective with a dipping lens, with the zoom set on 2. The tissue was illuminated laterally by two horizontal light sheets. Exposure time was 100 ms and the emitted fluorescence was collected at 690 nm. The step size used was 5 μm with voxel dimensions of 1.51 μm (x, y) and 5 μm (z). Images were processed as described in Supplementary Table 1A using the Imaris software (Bitplane V 9.0.1).

#### Osteocyte cytoskeleton imaging and analysis

Frozen bone samples (preparation B) were sectioned (100 μm) using a cryostat (CM3050S, Leica Microsystems) and were collected on SuperFrost Plus adhesion slides (Thermo Fisher Scientific). One section per animal was rinsed for 5 min with PBS 1X and permeabilized with 0.1% Triton X-100 for 1 h. A solution of phalloidin conjugated to Alexa 546 (3.3 μM, Invitrogen) in PBS 1X and 1% BSA was used to label osteocyte cytoskeleton overnight. Sections were mounted with Fluoromount-G (Invitrogen). Imaging was performed with a spinning disk confocal microscope (Leica DMi8 Yokogawa CSU-W1 + ILAS2) equipped with a 561 nm laser line (30%), and a 40× oil immersion objective. Exposure time was 200 ms. The step size used was 0.5 μm and the acquisition was performed on a total thickness of 20.5 μm. Voxel dimensions were 0.275 μm (x, y) and 0.5 μm (z). Images were processed as described in Supplementary Table 1B using the Imaris software (Bitplane V 9.0.1). From 60 to 150 osteocytes per animal were analyzed.

### Oxygen levels detection

A Leica CM 3050S cryostat was used for cryosectioning (Leica Microsystems, Nussloch, Germany). Ten microns frozen tissue sections (growing and mature groups from preparation A) were placed onto SuperFrost Plus slides (Thermo Fisher Scientific). Anti-EF5 antibody conjugated to Cyanine 5 (ELK3-51) staining was performed following the EF5 Hypoxia Detection Kit instructions. Briefly, sections were rinsed with PBS 1X and incubated overnight in a blocking solution with the recommended composition. After rinsing, sections were immersed in ELK3-51 antibody (75 μL/mL), 1.5% BSA solution for 4 h at room temperature. Finally, sections were incubated in DAPI (1 μg/mL, Thermo Fisher Scientific) for 5 min at room temperature to label the nuclei. Sections were rinsed and mounted with Fluoromount-G (Invitrogen). For negative control, sections from non-injected mice were incubated in ELK3-51 antibody solution. We assessed qualitatively the difference between the growing and mature groups as we mainly found labelled osteocytes in the growing animals, and the distribution of the fluorescent cells was non-homogeneous along the diaphysis.

### Immunolabelling of osteocyte proteins

#### Cryosections

A Leica (CM 3050S, Leica Microsystems, Nussloch, Germany) cryostat was used for cryosectioning. Ten-micron frozen tissue sections (cryostat CM 3050S, Leica Microsystems, Nussloch, Germany) were placed SuperFrost Plus slides (Thermo Fisher Scientific).

#### Immunofluorescence (IF) protocol

One section per animal was rinsed for 5 minutes with PBS 1X, permeabilized with 0.1% Triton X-100 for 10 min and then rinsed with PBS-T (0.2% Tween 20 in PBS 1X). The fixed sections were blocked with PBS-T containing 1% BSA, and 0.3 M glycine for 1 h at room temperature. The sections were then incubated with a dilution of primary antibody (Supplementary Table 2) in 1% BSA and PBS-T, overnight at 4°C. The sections were rinsed with PBS-T and then incubated with a secondary antibody (Supplementary Table 2) for 1 h at room temperature. Finally, sections were immersed in DAPI (1 μg/mL, Thermo Fisher Scientific) for 5 min at room temperature to label the nuclei. Sections were rinsed and mounted with Fluoromount-G (Invitrogen). Imaging was performed with a Leica TCS-SPE confocal microscope, using 40× or 63× oil immersion objectives, a zoom of 1.7, and a step size of 1 μm. Sections incubated in the secondary antibody alone were used as negative control for non-specific binding, the negative controls are provided in Supplementary Figure 4. Four pictures per bone section were analyzed using the Fiji software. For quantification, the number of total nuclei and positive cells was manually determined. The ratios of positive cell number over total cell number were calculated.

### Gene expression in osteocytes

#### Laser microdissection of osteocytes and mRNA extraction

We optimized and described the procedure of laser-assisted microdissection for cortical bone in Palmier et al. [20]. Briefly, five microns frozen tissue sections were collected on Frame slides (UV Technology, Carl Zeiss, Jena, Germany). Cell nuclei were stained with Cresyl Violet (Sigma Aldrich Co., St. Louis, MO, USA) and dehydrated in ethanol baths. The frame slide was assembled with a cover slip and the assembly was mounted on the laser microdissection device. Sections were microdissected using a 355 nm UV laser, coupled with an inverted microscope and software (P.A.L.M MicroBeam, Carl Zeiss, Jena, Germany). A total surface of 2.5 ± 0.1 mm^2^ was dissected (corresponding to approximately 1,500 osteocytes). Total RNA was extracted from microdissected tissue samples following the recommendations for MasterPure Complete DNA and RNA Purification Kit (Lucigen, Teddington, UK).

#### RT-qPCR

RNA was processed and analyzed according to an adaptation of published methods [44]. Briefly, cDNA was synthesized from total RNA by using qScript XLT cDNA SuperMix (Quanta Biosciences, Beverly, MA, USA). qPCR was performed with a LightCycler^®^ 480 Real-Time PCR System (Roche, Meylan, France). qPCR reactions were done in duplicate for each sample by using LightCycler 480 SYBR Green I Master (Roche) in a final volume of 10 μL. The qPCR data were exported and analyzed in the Gene Expression Analysis Software Environment developed at the Neurocentre Magendie (Bordeaux, France). Relative expression analysis was normalized against two reference genes tested as the most stable for the long bones: succinate dehydrogenase complex subunit (Sdha) and ribosomal protein 19 (Rpl19). To determine the reference genes, long bones from mice of different age groups were dissected, bone marrow was flushed, and RNA was extracted. RNA was processed as explained above. The Genorm method was used to determine the reference genes out of 10 candidates. The relative level of expression was calculated with the comparative (2^−ΔΔCT^) method. Primer sequences are reported in Supplementary Table 3.

### Statistics

Statistical analyses were carried out using GraphPad Prism 10. A non-parametric Kruskal-Wallis test (KW) was used, followed by Dunn’s multiple comparison test. *p* < 0.05 was considered as statistically significant. Spearman’s correlation and linear regressions were performed to test significant relationships between datasets. Spearman’s correlations were evaluated as strong (0.7–1.0), moderate (0.5–0.7), or weak (0.3–0.5).

### Data availability

The data that support the findings of this study are available from the corresponding author upon reasonable request.

## Supplementary Materials

Supplementary Figures

Supplementary Tables

Supplementary Video 1

Supplementary Video 2

Supplementary Video 3
