# Supplementary material for: Blood vessel and osteocyte networks concurrently rearrange during bone maturation and decline during aging in the femur of male mice
Source: Aging (Albany NY). 2025 Aug 22;17(8):2089–112. doi: 10.18632/aging.206302 (PMC12422823; doi:10.18632/aging.206302)
Supplement: Supplementary Figures [file aging-17-8-206302-s001.pdf]

SUPPLEMENTARY FIGURES

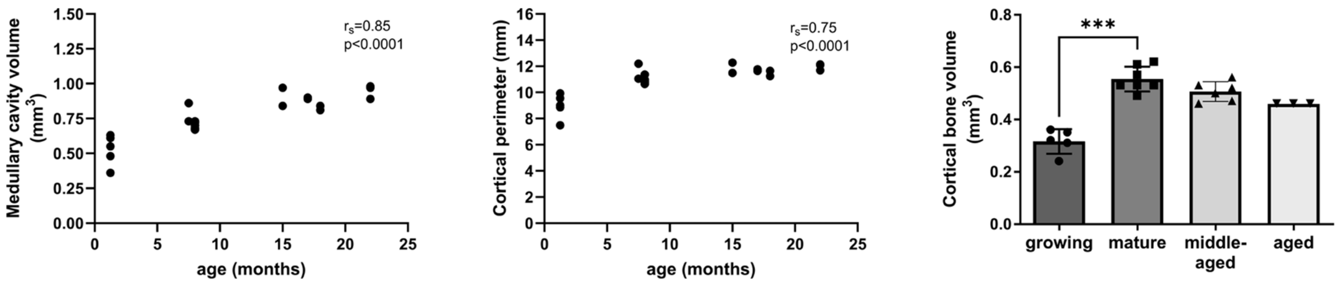

Supplementary Figure 1. MicroCT analysis of femur for the 4 age groups. Correlation between the medullary cavity volume and age, between the cortical perimeter and age, and comparison of the cortical bone volume between the 4 age groups. The data are presented as mean ± s.d. Statistical analyses: Spearman's correlation, KW, Dunn's multiple comparison test with the mature group as reference, $^{***}p < 0.001$.

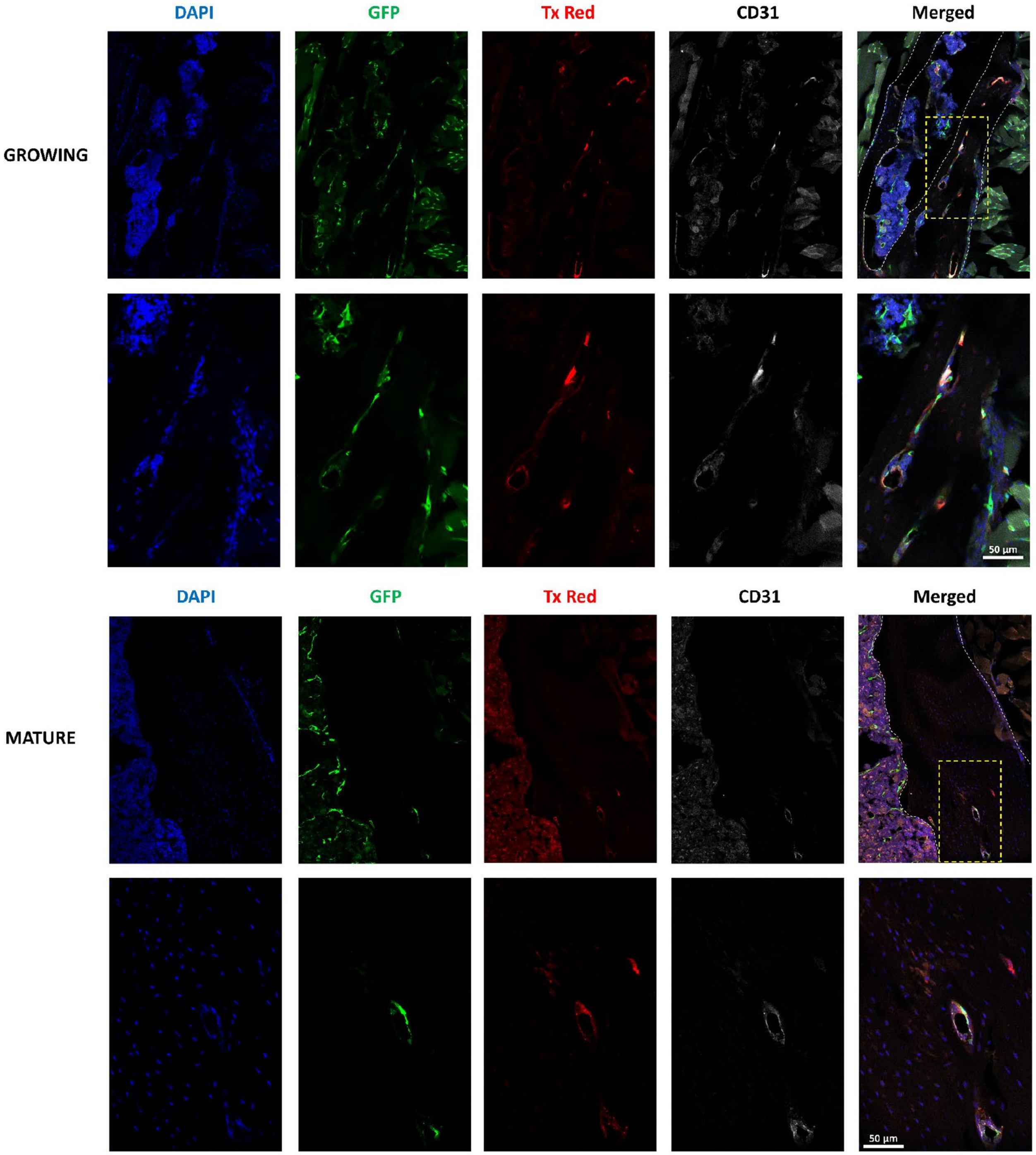
DAPI
GFP
Tx Red
CD31
Merged
GROWING
50 µm
DAPI
GFP
Tx Red
CD31
Merged
MATURE
50 µm

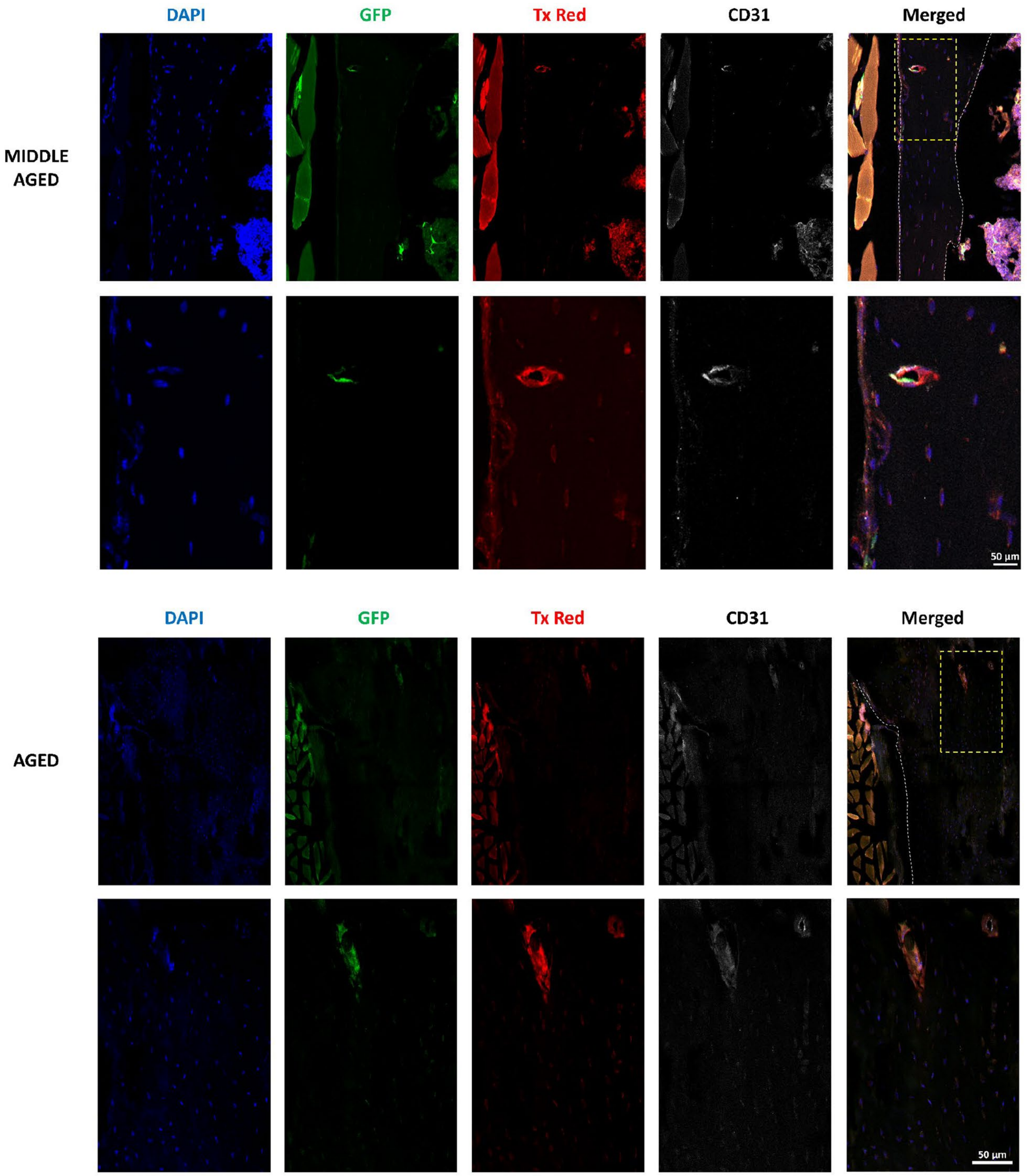
DAPI
GFP
Tx Red
CD31
Merged
MIDDLE AGED
50 µm
DAPI
GFP
Tx Red
CD31
Merged
AGED
50 µm

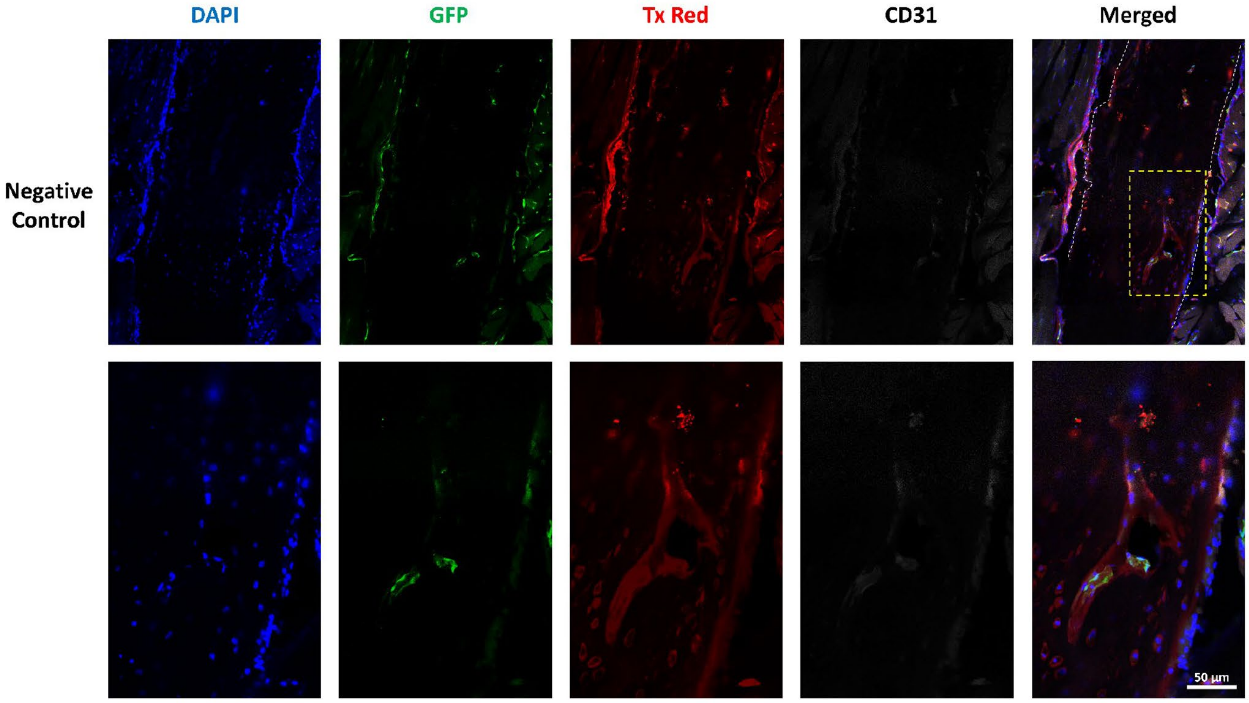

Supplementary Figure 2. Characterization of the blood vessels in the cortical bone. Images showing the co-localization of endogenous GFP, immunolabeling of CD31 in the blood vessels with diffused Texas Red-Dextran in the channels the cortical bone. DAPI in blue, GFP in green, Texas Red-Dextran in red, CD31 in white.

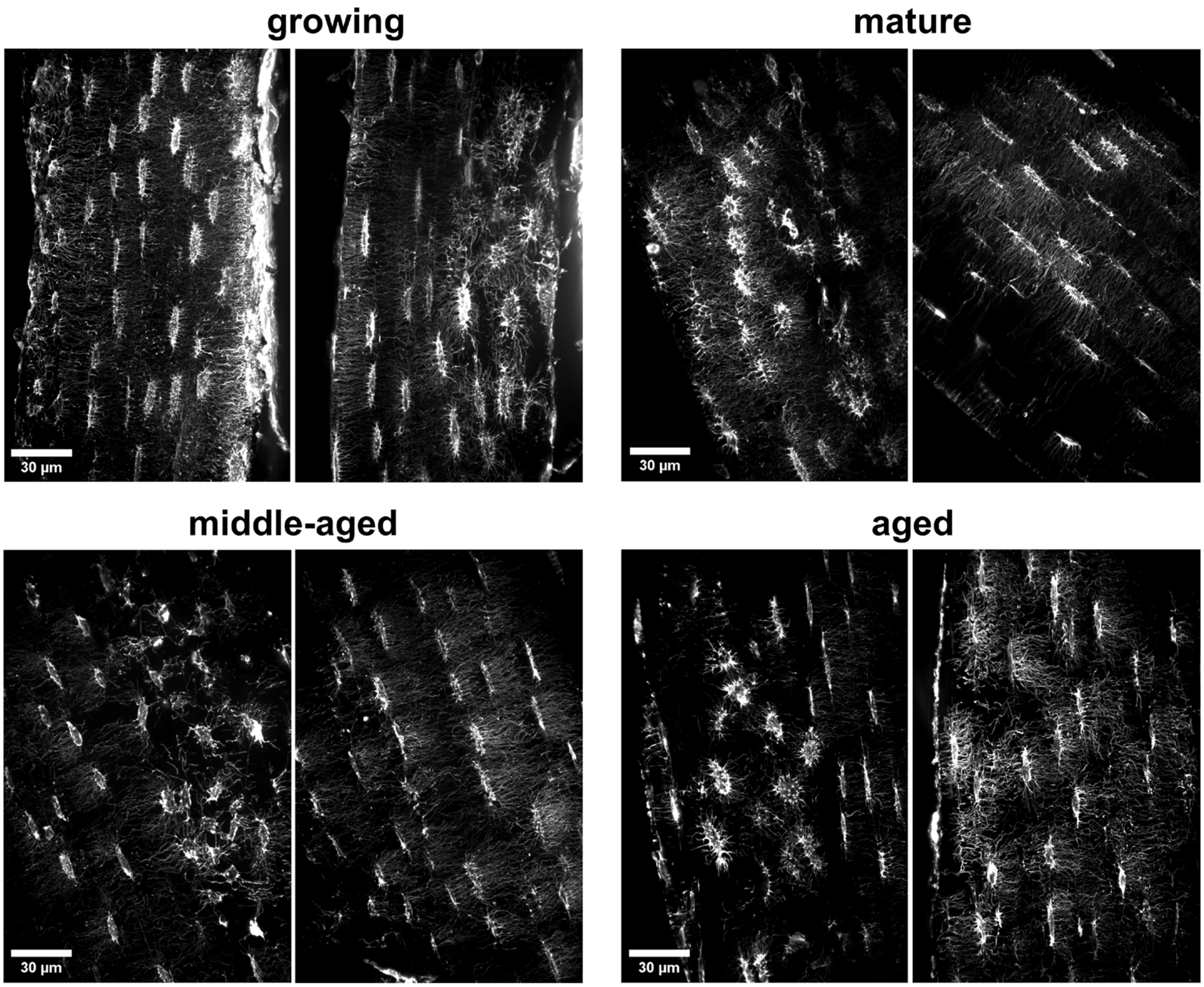

Supplementary Figure 3. Heterogeneity in the organization of the osteocyte network in the 4 groups studied. Phalloidin labeling in white.

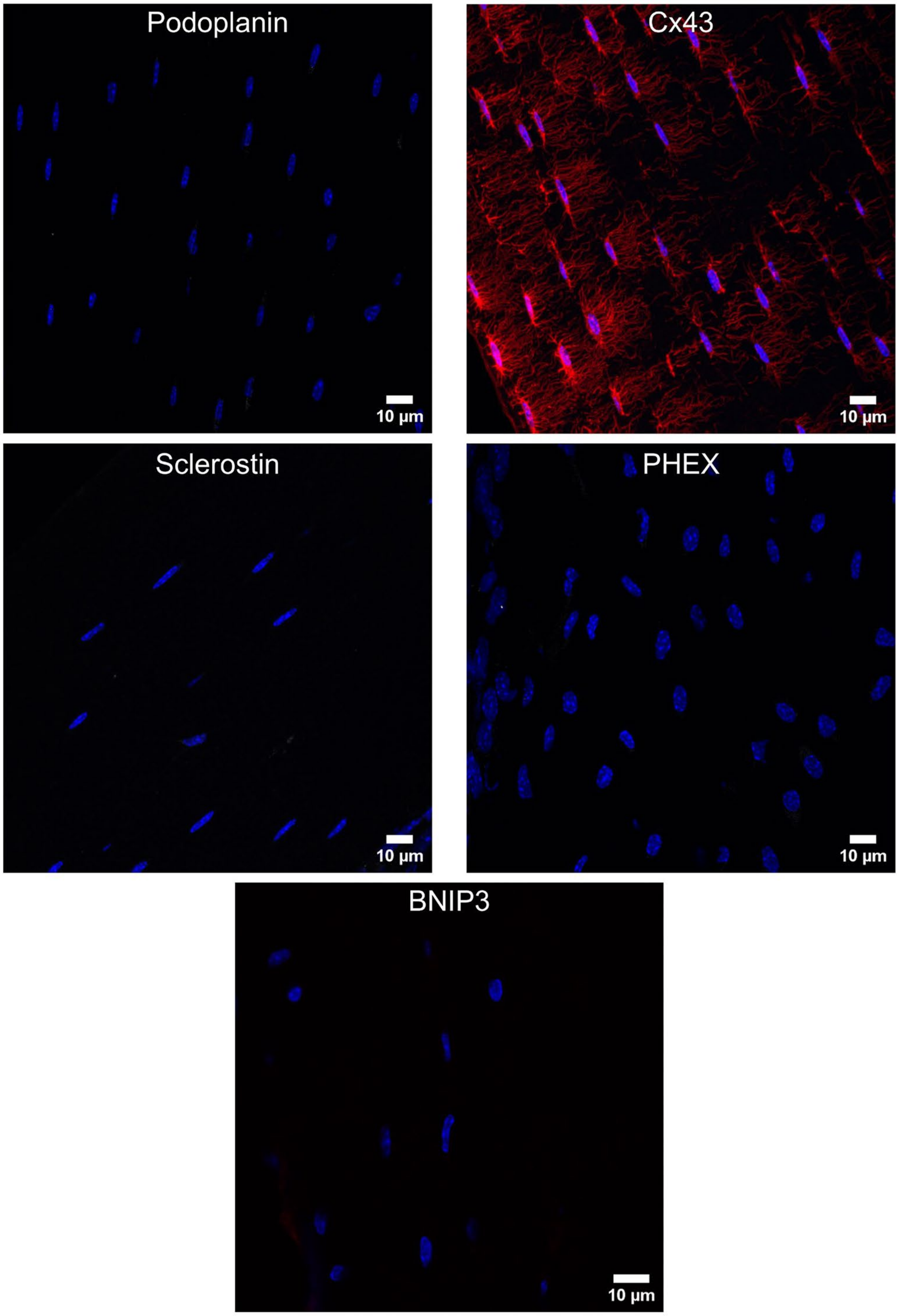

Supplementary Figure 4. Images of the negative controls for the Podoplanin, Cx43, Sclerostin, PHEX and BNIP3 immunofluorescences.
