# Supplementary material for: Blood vessel and osteocyte networks concurrently rearrange during bone maturation and decline during aging in the femur of male mice
Source: Aging (Albany NY). 2025 Aug 22;17(8):2089–112. doi: 10.18632/aging.206302 (PMC12422823; doi:10.18632/aging.206302)
Supplement: Supplementary Tables [file aging-17-8-206302-s002.pdf]

SUPPLEMENTARY TABLES

Supplementary Table 1. 3D image analysis protocol for the blood vessel (A) and osteocyte (B) networks.

Steps	Instructions for imaris software
**1A. Analysis of the blood vessel network**	
1	Use the manual surface mode to specifically delineate the cortical bone part with the help of masks.
2	Segment and measure the whole volume of cortical bone thanks to the automatic surface mode.
3	Create a new automatic surface to select manually lectin positive blood vessels inside the cortical bone and measure their total volume.
4	Calculate the ratio of the volume of blood vessels inside the cortical bone and the total volume of cortical bone.
**1B. Analysis of the osteocyte network**	
1	Crop rectangular volumes
2	Use the automatic surface mode in Imaris to determine the volume of the cropped image.
3	Use the Filament mode: autopath function with starting points adjusted at 2.5 µm and seed points at 0.6 µm.
4	Manually place the starting points inside the phalloidin labelled osteocyte bodies and count the number of cell bodies inside the volume.
5	Semi-automatically place the seed points along the dendrites. Seed points placed 10 µm around starting points were automatically removed to avoid filament creation inside osteocyte bodies.
6	Create filaments.
7	Automatically count the number of Scholl intersections 3 µm around the starting point inside de volume. It gives the estimated total number of dendrites inside the volume.
8	Divide the total number of dendrites by the total number of cell bodies to obtain the average number of dendrites per cell in the volume.

Supplementary Table 2. Primary and secondary antibodies.

Antibody	Catalog number, Company	Host species	Concentration
Anti-Connexin43	C6219, Merck	Rabbit	0.5 µg/mL
Anti-Sclerostin	AF1582, R&D System	Goat	7.5 µg/mL
Anti-Podoplanin	8.1.1: sc-53533, Santa Cruz Biotech	Syrian hamster	4 µg/mL
Anti-PHEX	BS-12313R, Bioss	Rabbit	20 µg/mL
Anti-BNIP3	ab109362, Abcam	Rabbit	13.53 µg/mL
Anti-CD31	BD553370, BD Biosciences	Rat	5 µg/mL
Donkey anti-goat Alexa 647	A-21447, Invitrogen	Donkey	2 µg/mL
Goat anti-rabbit Alexa 647	A-21245, Invitrogen	Goat	2 µg/mL
Goat anti-syrian hamster Alexa 647	ab180063, Abcam	Goat	2 µg/mL
Donkey anti-rat Alexa 647	ab150155, Abcam	Donkey	2.5 µg/mL

Supplementary Table 3. Primer sequences.

Gene	GenBank ID	Forward sequence (5′−3′)	Reverse sequence (5′−3′)
Rpl19	NM_009078	GGCTCGTTGCCGGAAAAACA	TCGTCCTTCTCATCCAGGTCA
Sdha	NM_023281	TACAAAGTGCGGGTCGATGA	TGTTCCCCAAACGGCTTCT
Gja1	NM_010288	ACTCTCCTTTTCCTTTGACTTC	TGGACCTTGTCCAGCAGCTT
Pdpn	NM_010329	CGTCTTGTTAGCCATTGGCTT	GGCAAGTTGGAAGCTCTCTTA
Dmp1	NM_001359013	TGAAGAGAGGACGGGTGATTT	CCCAGCTCCTCTCCAGATT
Sost	NM_024449	TCCTGAGAACAACCAGACCAT	GCGGCATGGGCCGTCTGT
Phex	NM_011077	CAATTCCTATAGACCAGAAGCT	AGTGGAATTTCGTGGACAGTTA
Vegfa	NM_001025250	GCAAGGCGAGGCAGCTTGA	TTCCGGTGAGAGGTCTGGTT
